# Effects of salbutamol on airway characteristics in mechanically ventilated adults without COPD

**DOI:** 10.1186/cc10709

**Published:** 2012-03-20

**Authors:** J Van Rosmalen, QL Habes, I Havinga, F Van Beers, A Van Hees, D Ramnarain, JA Van Oers

**Affiliations:** 1 St Elisabeth Hospital, Tilburg, the Netherlands

## Introduction

In our ICU, salbutamol inhalation to prevent bronchospasm is standard care in mechanically ventilated (MV) patients. In MV patients without COPD the effect of salbutamol remains unclear. Therefore we examined the effect of inhaled salbutamol on resistance and compliance in MV patients without COPD.

## Methods

In this prospective study, we enrolled 11 critically ill MV patients without COPD. These intubated patients were on volume-controlled ventilation (6 ml/kg/PBW). Exclusion criteria were the use of β-blockers, propofol or neuromuscular blockers. They received five puffs of salbutamol (100 μg/puff ) delivered by metered dose inhaler via the adapter on the Y-piece. Ventilator settings and body position were unchanged during the study. Before and after salbutamol administration vital signs were recorded and lung mechanics were measured using the ventilator (Servo-i^® ^or Hamilton-G5^®^) at -1, +1, +15, +30, +60, +90 and +240 minutes. Values after administration of salbutamol (T0) were compared to those before administration. Results are presented as mean ± SD. Data were evaluated by paired *t *test and *P *< 0.05 was taken as statistically significant.

## Results

The study group consisted of seven men and four women, mean age 53 years. Underlying causes for ventilation were diverse. The median time spent on the ventilator before inclusion was 36 hours (6 to 151). After salbutamol administration inspiratory resistance and dynamic compliance decreased, but not significantly. Expiratory resistance, dynamic compliance, elastance, SpO_2 _and EtCO_2 _did not change (Table 1).

**Table 1:** 

	T-1	T0	T+1	T+15	T+30	T+60		T+90	T+240	
R_ins _(cm H_2_O/l/second)	14 (6)	Salbutamol administration	14 (4)	13 (5)	13 (5)	12 (4)		12 (5)	12 (4)	NS
R_exp _(cm H_2_O/l/second)	17 (7)		17 (6)	16 (6)	17 (8)	18	(10)	18 (8)	17 (7)	NS
C_dyn _(ml/cm H_2_O)	44 (12)		42 (11)	42 (11)	37 (10)	36 (9)		38 (9)	38 (8)	NS
C_stat _(ml/cm H_2_O)	49 (13)		49 (12)	47 (13)	43 (11)	44 (12)		45 (12)	43 (12)	NS
Elastance (cm H_2_O/l)	19 (3)		20 (4)	21 (4)	22 (4)	23 (4)		21 (4)	18 (8)	NS
Heart rate (beats/minute)	81 (22)		84 (24)	85 (23)	87 (22)	85 (22)		82 (18)	84 (20)	NS
SpO_2 _(%)	98 (1)		98 (2)	98 (2)	98 (1)	98 (2)		98 (2)	97 (2)	NS
EtCO_2 _(kPa)	4.7 (0.7)		4.7 (0.7)	4.7 (0.7)	4.7 (0.7)	4.7 (0.7)		4.7 (0.8)	4.7 (0.7)	NS

## Conclusion

There was no significant effect of salbutamol inhalation on airway characteristics and vital signs in non-COPD patients on MV. Therefore standard salbutamol inhalation in MV patients without COPD can be aborted.
